# Nutrients and Porphyria: An Intriguing Crosstalk

**DOI:** 10.3390/ijms21103462

**Published:** 2020-05-14

**Authors:** Elena Di Pierro, Francesca Granata

**Affiliations:** Fondazione IRCCS Ca’ Granda Ospedale Maggiore Policlinico, UOC General Medicine, via F. Sforza 35, 20122 Milan, Italy; francesca.granata@policlinico.mi.it

**Keywords:** porphyria, nutrients, nutrigenomics, lipid and glucose metabolism, genetic factors, diet, personalized nutrition, iron metabolism

## Abstract

Porphyria refers to a group of fascinating diseases from a metabolic and nutritional standpoint as it provides an example of how metabolic manipulation can be used for therapeutic purposes. It is characterized by defects in heme synthesis, particularly in the erythrocytes and liver. Specific enzymes involved in heme biosynthesis directly depend on adequate levels of vitamins and minerals in the tissues. Moreover, micronutrients that are required for producing succinyl CoA and other intermediates in the Krebs (TCA) cycle are indirectly necessary for heme metabolism. This review summarizes articles that describe the nutritional status, supplements intake, and dietary practices of patients affected by porphyria, paying special attention to the therapeutic use of nutrients that may help or hinder this group of diseases.

## 1. Introduction

**Figure 1:**
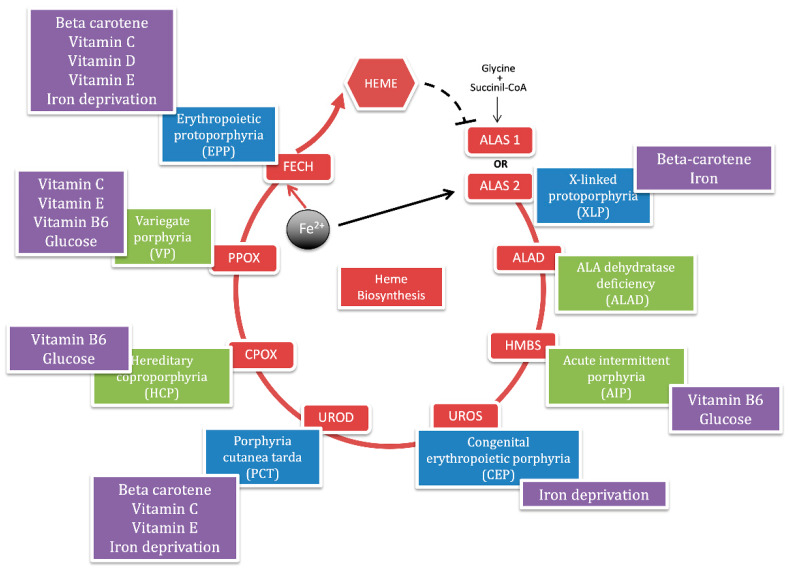
Heme biosynthetic pathway, porphyrias and nutrients.

Porphyria is a group of hereditary metabolic defects in heme biosynthesis that involves eight functionally interlocked enzymes required for the conversion of glycine and succinyl-coenzyme A to heme [1]. Heme is required for a variety of hemoproteins that are synthesized in response to metabolic needs. The hemoglobin synthesis in erythroid precursor cells accounts for about 85% of the daily heme synthesis in humans. Hepatocytes account for most of the rest, primarily for synthesis of cytochrome P450 enzymes [2]. In erythroid cells, the synthesis of heme is regulated, during erythroid differentiation, by erythropoietin and iron availability. In the liver, the heme synthesis is under a negative feedback regulation by the intracellular uncommitted heme pool [3] (Figure 1).

The selective blockage at specific enzymatic steps results in the overproduction and accumulation of heme intermediates such as 5-aminolevulinic acid (ALA), porphobilinogen (PBG) and uro, copro, or proto porphyrins. The degree of overproduction of intermediates is dependent on the degree of stimulation of the system by endogenous or exogenous stressors that induce the housekeeping and erythroid-specific 5-aminolevulinate synthase enzymes (ALAS-1 and ALAS-2), which catalyze the first step in heme biosynthesis [4].

Each type of porphyria has a unique pattern of accumulation and is associated with characteristic clinical features. Evidence suggests that the porphyrin precursors (ALA and PBG) cause symptoms by injuring neurons, while the porphyrins cause symptoms by injuring the skin [5]. The porphyrias are classified as acute if patients present with neurovisceral acute attacks and as cutaneous if they present with blistering or photosensitivity of the skin.

Also, porphyrias are classified as hepatic or erythropoietic according to the major site of expression of enzyme deficiency and overproduction of heme precursors [6]. The term of acute hepatic porphyria (AHP) is used for four disorders: ALA dehydratase deficient porphyria (ADP), acute intermittent porphyria (AIP), hereditary coproporphyria (HCP), and variegate porphyria (VP), although HCP and VP can also be associated with only skin lesions. Porphyria cutanea tarda (PCT) is a hepatic cutaneous porphyria. While the group of erythropoietic cutaneous porphyria (ECP) includes the common and the rare X-linked form of erythropoietic protoporphyria (EPP and XLP), congenital erythropoietic porphyria (CEP) and the hepatoerythropoietic porphyria (HEP), which is the homozygous recessive form of PCT [7] (Figure 1).

The red circle represents the heme biosynthetic pathway with the abbreviations of each enzyme in the red boxes. The iron is internalized in the porphyrin ring by ferrochelatase enzyme (FECH) to give heme, which negatively regulates the *ALAS-1* gene in hepatocyte (black dotted line). The black arrow points out the iron positive feedback on the *ALAS-2* gene in erythrocytes. Each enzyme is assigned with a panel that reports the corresponding porphyria. The blue panels represent the cutaneous porphyrias and the green panels represent acute porphyrias. In the purple boxes, we summarize nutrients (glucose); micronutrients (vitamin B6, C, D, E, and Beta-carotene) and minerals (iron) that are described in this review for each form of porphyria.

## 2. Diet

Although enzyme deficiencies in acute hepatic porphyrias are usually inherited, the enzyme deficiencies alone do not cause the disease. Additional factors that lead to a critical deficiency in the regulatory pool of heme within hepatocytes determine the disease manifestations [8]. Moreover, it is widely accepted that increased excretion of ALA and PBG is not necessarily correlated with symptoms even though excretion of these metabolites is further increased during an acute attack. Also, a marked fluctuation in the excretion of ALA and PBG from day to day is found in symptomatic and asymptomatic AHP patients. 

A stabilization in urinary levels of these metabolites was reported in AIP patients following a diet regimen that included a balanced content of protein, fat, and carbohydrate to maintain weight [9]. Moreover, the same author reported that the reduced calorie intake to 60–80 percent and the isocaloric substitution of fat for protein alone or for protein together with carbohydrate were associated with increased excretion of both porphyrin-precursors. Considering the marked decrease in PBG excretion due to the addition of carbohydrate in the diet, they concluded that the fluctuation in the excretion of porphyrin-precursors is related to protein and carbohydrate intake rather than total caloric content per se. 

Interestingly, increased calorie intake has been reported to be negatively correlated with urinary PBG levels in AIP patients suggesting that also the total energy intake affects the biochemical disease activity [10].

Recently, nutritional assessments of VP and AIP patients have been carried out using validated questionnaires that included a 24 h recall during 7-day dietary record [11]. No significant differences were observed in the nutrient intake and food pattern between symptomatic patients compared to asymptomatic carriers of VP [12]. Moreover, no difference in sugar, candies, or slow-release carbohydrate foods intake was found between the asymptomatic and symptomatic AIP cases [10]. On the contrary, significantly lower intakes of lipids, saturated fatty acids (SFA) and monounsaturated fatty acids (MUFA) were detected in AIP patients than in matched controls [13]. However, energy, protein, carbohydrate, polyunsaturated fatty acids (PUFA), cholesterol, and daily fiber intakes were reported to be similar in both groups [13]. 

Despite this, it has been reported that hypercholesterolemia may be present in AHP patients although without atherogenic potential [14]. In order to improve the results of a diet study, it would be useful to use the gold standard method such as DEXA (dual-energy X-ray absorptiometry) for the calculation of basic metabolism and a direct approach through expert nutritional assessment [15]. In summary, the changes in diet can alter the excretion of porphyrin-precursors in AHP patients. However, especially considering the lack of toxicity of ALA and PBG [16], it should not be concluded that changes in diet are capable of affecting the clinical disease activity.

## 3. Glucose

The therapeutic effect of glucose in patients with AHP is well documented [17,18]. Carbohydrate loading is widely used not only for the treatment but also in the prophylaxis of the acute attack. Moreover, fasting is one of the precipitating factors in the AHP crises [19]. The addition of glucose to cultured chick embryo hepatocytes and the carbohydrate feeding in rats have been first reported to cause a concentration-dependent impairment of drug-mediated induction of ALAS-1 enzyme [20,21].

Successively, Handschin et al. revealed that PGC-1α (peroxisomal proliferator-activated cofactor 1α), a transcriptional co-activator of nuclear receptors and other transcription factors, is a key player in both porphyria induction via fasting and amelioration of symptoms by glucose treatment [22]. Using mice with a liver-specific deficiency in PGC-1α and isolating primary hepatocytes, they demonstrated the ability of this factor to increase markedly the production of *ALAS-1* mRNA. Furthermore, the authors also established the effect of glucose loading in reducing the *ALAS-1* transcript levels 30 min after injection. The combination of glucose and insulin was more potent in inhibiting fasting-mediated induction of PGC-1α and ALAS-1, supporting the hypothesis that at least part of the beneficial effect of glucose in AHP attacks is mediated by the glucose-triggered increase of plasma insulin.

**Figure 2:**
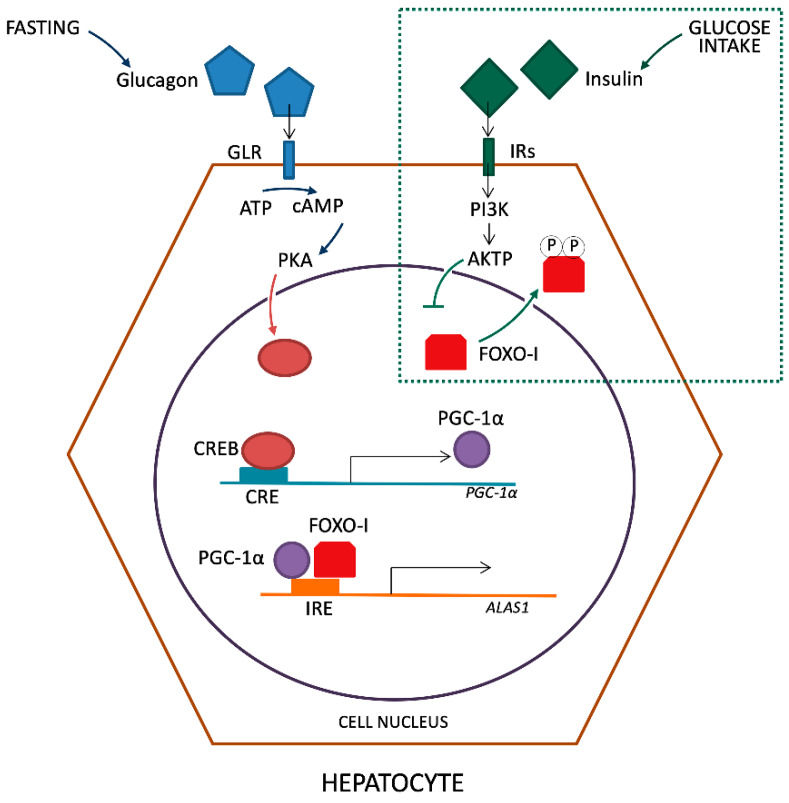
Regulation of ALAS-1 transcription by glucagon and insulin.

The induction of PGC-1α during fasting is due to glucagon action on the cAMP response element-binding transcriptional factor (CREB), which binds directly to the *PGC-1α* promoter [23]. Also, the direct activation of the *ALAS-1* promoter by CREB was described [24]. Moreover, it has been established that the insulin pathway involving the protein kinase B (Akt) in the liver inhibits *ALAS-1* transcription [25]. It is also known that the activated Akt, in turn, phosphorylates the transcriptional factor FOXO1 (Forkhead box protein O1) disrupting its binding to PGC-1α [26,27]. Then, it was believed that the activation of *ALAS-1* expression by PGC-1α is due to the co-activation of FOXO1, which binds to the insulin-response element in the promoter of *ALAS-1* and this interaction can be disrupted by insulin signaling [28] (Figure 2).

However, discordant data have been reported on this aspect. An in vivo study indicated that the serum glucose level was unchanged, but fasting insulin levels were higher and the glucagon was lower in mice with AIP than in the wild-type mice [29]. Moreover, the drug-mediated induction of ALAS-1 significantly disturbs the hormonal status that regulates carbohydrate metabolism by increasing insulin levels while decreasing glucocorticoids synthesis, metabolization, and plasmatic levels in animal models [30]. Also, the finding that AIP patients with acquired type 2 diabetes mellitus did not longer have symptoms of AIP supports the protective role of elevated blood glucose levels [31]. On the contrary, symptomatic AIP patients showed decreased insulin release and C-peptide levels in plasma associated with increased disease activity, indicating that a decreased glucose uptake by cells may explain accelerated heme synthesis [32]. Fasting insulin is lower, and the glucose/insulin ratio is higher in AIP patients with high urinary PBG levels than in patients with low urinary PBG levels [10].

At the same time, the screening of serum hepatic proteins revealed that a significant number of AIP patients presented a decrease in insulin-like growth factor 1 IGF-1, transthyretin (prealbumin) or both [33]. Due to its structural similarity with insulin, IGF-1 interacts with insulin receptors and has insulin-mimicking effects. Besides, transthyretin and IGF-1 are useful markers for predicting nutritional status and are known to decrease during inflammation or liver disease [34,35,36]. The decrease in transthyretin and IGF-1 levels in AIP patients therefore could reflect a metabolic disturbance restricted to the liver and/or the existence of chronic liver inflammation. A higher resistin level in the symptomatic than in the asymptomatic AIP patients and its positive correlation with leptin levels may indicate that inflammation, adipokines, and hormones affecting insulin resistance may be involved in higher disease activity [10]. However, most inflammatory biomarkers and cytokines were not correlated with ALA, PBG or porphyrin levels [32].

Conversely, accelerated protein degradation, decreased rate of synthesis of liver proteins and increased amino acid catabolism and nitrogen loss may be secondary to inflammatory disease because of increased metabolic demands [37]. Thus, a change in liver energy metabolism in AIP patients could support the induction of ALAS-1, thereafter, worsening the symptoms of the disease and contributing to the persistence of the clinical manifestations. It was also found that liver graft recipients from an AIP patient developed AIP symptoms and increased PBG levels [38] and that liver transplantation in severe AIP and VP patients normalizes the excretion of ALA and PBG [39,40] confirming the importance of the liver in the pathophysiology of this disease.

The activation of PGC-1α takes place through fasting and glucagon release that links its receptor (GLR) with consequent ATP reduction by adenylate cyclase in cAMP in the cytosol of hepatocytes. In turn, it leads to the activation of protein kinase A (PKA). In the nucleus of hepatocyte, the active form of PKA leads to the activation of transcription factor CREB that links the active site of CRE on *PGC-1α* gene promoter. The PGC-1α and FOXO-I bind together with the insulin responsive element (IRE) site on the *ALAS-1* gene promoter, inducing transcription. The introduction of glucose and subsequent production of insulin, detected from membrane Insulin Receptor (IRs), lead to the phosphorylation of FOXO-I by the intervention of PI3K and AKTP. Phosphorylated transcriptional factor FOXO is carried out of the nucleus inhibiting the synergistic activation with PGC-1α on the *ALAS-1* promoter.

## 4. Iron 

Iron is classified as a nutritional and fundamental microelement required for oxygen transport, electron transfer, oxidase activities, and energy metabolism. The major intake of iron is through the diet. Foods that contain a relatively high concentration of iron include meat, fish, cereals, beans, nuts, egg yolks, dark green vegetables, potatoes, and fortified foods. The median dietary intake of iron in adults ranges between 9.4 and 17.9 mg/day (higher in males compared to females) with the average basal iron loss ranges 0.95–5.9 mg/day in men and 1.34–7.4 mg/day in women. Nutrient composition data for iron are derived from the European Food Safety Authority (EFSA) Nutrient Composition Database [41].

Iron has a central role in heme biosynthesis and also in erythropoietic cutaneous porphyria due to the positive feedback between iron and ALAS2, the first enzyme of erythroid heme biosynthesis. Moreover, it serves as a substrate of the ferrochelatase enzyme in the last step of the heme pathway. Iron deficiency can occur in EPP and XLP patients [42]. While iron overload is reported in PCT (in 90% of patients) [43,44] and CEP (all patients) [45]. The presence of hepatic iron in PCT patients has been associated with hemochromatosis (HFE) gene mutations, hepatitis C (HCV), and human immunodeficiency (HIV) viral infections. The presence of hepatic iron generates a UROD inhibitor, uroporphomethene, identified in the human liver biopsies of patients with PCT [46]. 

The presence of intracellular iron also has a strong impact on the cellular redox status leading to an increase in oxidative status in these patients. Interestingly, a study on the dietary intervention was performed on a group consisting of 13 male PCT patients to decrease iron overload. Patients were evaluated for different parameters including serum iron level, before and after three weeks of the vegetable–fruit diet, and its daily caloric content was ca. 500 kcal/day. The results showed a significant decrease in iron and ferritin levels after dietary caloric restriction [47]. Given that, dietary caloric restriction could be used in support of phlebotomy (specific therapy for the reduction of porphyrins in the blood) in order to reduce iron overload as suggested in the latest review on PCT [48]. Future studies on the reduced intake of iron-rich foods should be carried out.

Iron overload in CEP patients is due to ineffective erythropoiesis that characterizes this disease and blood transfusion therapy. Dietary iron restriction in these patients has not been described because the large amount of this mineral is not ameliorable with a diet but only with iron-chelation and phlebotomy [49,50].

It should be emphasized that there is no well-designed clinical trial that can establish the role of iron supplementation in EPP and XLP patients. Moreover, while the iron is a substrate of ferrochelatase (FECH), the defective enzyme in the EPP, it is the limiting substrate in XLP caused by a gain of function of ALAS2. Therefore, the response of oral supplementation could be different in the two forms of protoporphyria. Only one case of XLP was reported in which iron substitution increased hemoglobin concentration and decreased concentrations of both protoporphyrin IX (PPIX) and zinc protoporphyrin (ZnPP). Hence, iron supplementation could be useful for XLP patients with mild microcytic anemia [51].

Regarding EPP, the mechanism of iron deficiency is not completely understood, and the benefit of iron supplementation is controversial [52] despite the strong evidence that confirms the benefit of mild anemia on photosensitivity symptoms in EPP patients [53,54]. In the literature search, six studies have shown that oral iron produced biochemical (an increase of PPIX) and clinical (increasing of photosensitivity) worsening of symptoms in EPP patients [55,56,57]. By contrast, bibliographic research found two cases that described a reduction in photosensitivity during iron supplementation [58]. 

In summary, in the presence of in vitro tests on the improvement of the clinical symptoms in anemia condition, but in the absence of clinical trials that also exclude the placebo effect, it is necessary to go deep on this controversial aspect in patients affected by protoporphyria, to realize a personalized therapy. It is important to underline that in six clinical cases reporting a negative effect of iron, patients also had a slightly compromised liver condition. Only the study of these patients will lead to the formulation of an adequate oral supplementation followed by a control regarding the dietary intake of this important vital microelement.

## 5. Vitamins

### 5.1. Vitamin B6

Vitamin B6 (pyridoxine) is a water-soluble vitamin that is important for the normal functioning of multiple organs. Vitamin B6 is found mainly in animal foods (meats, fishery products, offal, etc.) as well as in vegetables (whole unprocessed cereals, legumes, oilseeds, etc.). Vitamin B6 deficiency is quite rare and is associated with other avitaminosis or hypovitaminosis [59] (Table 1). It is metabolized to pyridoxal-5-phosphate (PLP), an active molecule, which serves as a coenzyme for more than 100 enzyme reactions including neurotransmitter production, protein, glucose, lipid, and amino acid metabolism [60]. Moreover, PLP is directly involved in the first step of heme synthesis as a cofactor of ALAS, the rate-limiting enzyme for the formation of ALA [61]. 

Experimental vitamin B6 deficiency was induced in an asymptomatic AIP patient with high excretion of heme precursors employing a synthetic diet and administration of deoxypyridoxine, a pyridoxine antagonist drug [62]. The authors reported that ALA and PBG levels declined significantly during vitamin B6 deficiency and increased again following the administration of vitamin B6 without other alterations in the diet. Interestingly, a significant deficiency of vitamin B6 in the plasma and erythrocytes was reported in patients with AIP and VP [63]. 

Also, the mitochondrial concentration of glycine, which is the first substrate in heme biosynthesis was found to be inversely proportional to vitamin B6 intake [64]. Different PLP-dependent enzymes are involved in glycine synthesis and catabolism, including glycine transaminase that catalyzes the transformation of glyoxylate to glycine preventing oxalate formation [65]. In patients with AIP and VP, significantly elevated levels of oxalic acid (OA) in plasma and urine were also observed concomitantly with vitamin B6 deficiency [63]. 

Through indirect evidence, Elder and Mengel suggested that an excessive metabolic requirement and use of vitamin B6 in mitochondrial heme synthesis could cause relative intracellular vitamin B6 deficiency in patients with AIP reducing the activity of cytoplasmatic PLP-dependent enzymes [62]. Increased homocysteine levels in symptomatic AIP patients were also reported suggesting that the consumptive depletion of PLP due to increased demand by ALAS hyperactivity can also impair the detoxification of homocysteine [66]. 

However, severe vitamin B6 deficiency did not impair the induction of hepatic ALAS-1 in starved animals demonstrating that the hyper activation of ALAS-1 is unlikely to produce significant depletion of plasma PLP [67]. Then, low plasma levels of PLP reported in AHP patients are probably the result of factors other than increased enzyme levels. However, further extensive studies are necessary to establish the relationship with the disease activity. 

### 5.2. β-. Carotene

Β-Carotene, best known as provitamin A, is the most common reddish-orange plant pigment with fat-soluble characteristics and high levels of bowel absorption. Previous studies have shown that oral supplementation of β-carotene has some effect on human health due to its antioxidant proprieties. Many studies describe the role of β-carotene in photo-protection and its effectiveness in preventing UV-induced erythema in a healthy population [68] (Table 1).

Due to these properties, from 1970 to 2004, β-carotene was used for cutaneous porphyria, especially on EPP patients [69,70,71]. Minder et al. in 2009 analyzed a majority of articles on β-carotene from 1972 to 1996 and summarized 16 studies on oral administration of β-carotene [72]. Of these, one was a randomized controlled trial, while 15 studies were open-labeled uncontrolled studies, including 12 retrospective case reports. In all studies, the efficacy criteria were not standardized. The dose ranged from 100 to 300 mg/day in adults and from 30 to 90 mg/day in children besides normal food intake. The authors concluded that the results were strongly contradictory, and efficacy was inversely correlated with study quality.

Oral Lumitene™ (β-carotene) (120–180 mg/day) has been used to improve tolerance to sunlight in XLP, but the available data are insufficient to prove the efficacy of the treatment [73,74,75]. Also, experiments on chicken eggs exposed to PPIX, irradiated with ultraviolet A (UVA), and treated with β-carotene or melanin were reported in the literature. Β-carotene treated eggs showed four times more mortality compared with melanin treated eggs suggesting the lack of efficacy of β-carotene [76]. In conclusion, there is no scientific evidence that proves the efficacy of β-carotene in EPP and XLP patients even though in some of these case reports, an improvement in photosensitivity has been reported at high doses of β-carotene. 

The reduction of carotenoids in the serum of PCT patients was also described demonstrating that increased use of these molecules occurs in this form of porphyria [77]. PCT is characterized by high levels of porphyrins production and iron overload that are responsible for a synergistic effect in intracellular oxidative damage. Thus, the depletion of antioxidative liposoluble molecules supports the hypothesis that the beneficial effects of β-carotene may involve quenching of singlet oxygen or free radicals. 

Although the application of β-carotene has long been used as a likely therapy, the use of this nutrient has disadvantages. In 2012, the European Food Safety Authority (EFSA) published a systematic literature review and meta-analysis on the benefit and toxicity of β-carotene as a nutritional supplement [78]. The analysis shows nine randomized controlled trials that demonstrated an increased risk of lung and stomach cancers, particularly in smokers and asbestos workers at dose levels ≥ 20 mg/day. Therefore, the EFSA experts concluded that for the safe use, the dose should not exceed 25 mg/day in humans.

Considering this, the β-carotene doses used in EPP, XLP, and PCT patients, that is, 100 mg/day to 300 mg/day could be dangerous to human health. Moreover, it is consolidated from the latest scientific evidence that a single supplement is not as effective as the integration and interaction of multiple antioxidant nutraceuticals through a balanced diet [79].

### 5.3. Vitamin E

Vitamin E or alpha-tocopherol is a lipid-soluble vitamin with antioxidant proprieties closely associated with vitamin C and glutathione reductase (GR). In particular, vitamin C regenerates the reduced form of vitamin E, which utilizes GR to convert the oxidized form of glutathione (GSSG) into its reduced form (GSH) useful to reduce superoxide anion (O2•−) and to restore oxidative balance [79]. Plants naturally synthesize Vitamin E, which is contained in nuts, plant seeds and plant oils. Vitamin E absorption requires the presence of fat [80] (Table 1).

The chronic accumulation of heme intermediates in erythrocytes, liver, and other cell types can induce cellular damage due to their ability to produce free radicals and activate oxygen leading to oxidative stress [81,82]. 

Antioxidant defenses and oxidative stress have been studied in some types of porphyria. Decreased plasma antioxidant vitamin levels and increased oxidative damage markers have been described in patients with PCT [77,83]. Moreover, a significantly reduced activity of antioxidant enzyme catalase (CAT) and GR has been reported in neutrophils of VP patients with increased levels of plasma malondialdehyde (MDA), a marker of lipid peroxidation [84]. By contrast, no differences have been found in the levels of antioxidant vitamins or oxidative damage markers in AIP patients [85].

In light of this evidence, the efficacy of vitamin E supplementation in cutaneous porphyria patients can be easily understood. Also, Vitamin E is able to interact with the enzyme uroporphyrinogen decarboxylase involved in PCT to increase its activity. Patients treated with alpha-tocopherol (1 g/day) for one month showed a reduction in urinary porphyrins than that of untreated patients [86]. Szekely E. et al. performed a randomized controlled study on 23 PCT patients treated with 200 mg/day of alpha-tocopherol acetate in conjunction with phlebotomy for eight weeks and demonstrated a decrease in porphyrins and an increase in sunlight exposure [87]. In both studies, the measurement of parameters related to oxidative stress after supplementation was not performed. 

A double-blinded crossover study was also conducted on women affected by VP. Dietary supplementation with vitamin E (50 mg/day) and vitamin C (150 mg/day) for six months ameliorated oxidative stress with a reduction in plasma MDA levels and induced the activity of CAT and GR in erythrocytes. However, no effects of supplementation were observed on oxidative damage markers or antioxidant enzyme activities in neutrophils [84]. Conversely, treatment with antioxidants failed to produce beneficial results in AIP patients. As indicated by the biochemical and clinical variables monitored, neither the incidence of porphyric crisis, not the severities of the attacks were affected by the antioxidants administered [88]. 

The serum level of vitamin E was reported significantly lower in PCT patients than in healthy controls, particularly in patients with a liver injury [89]. Lower levels of vitamin E were also identified in the serum of EPP patients, and it was observed that the vitamin inhibited the in vitro photohemolysis of red blood cells [90,91]. After this discovery, there was only one case report on vitamin E treatment in a 27-year-old EPP man with liver cirrhosis. Following the administration of vitamin E, the concentration of protoporphyrin in erythrocytes decreased significantly, and the liver function tests were improved, although there is no clear evidence of its role in EPP patients [92].

It is widely accepted that nutrient supplementation, along with a balanced and healthy diet, can be used to improve the general health of patients with chronic illnesses [68,79]. It is believed that patients with PCT, VP, and EPP may request for these supplements to increase their antioxidant capacity and to address oxidative stress due to chronic accumulation of porphyrins.

### 5.4. Vitamin C

Vitamin C exists in plasma in its reduced form as ascorbic acid. Ascorbic acid is an essential nutrient with powerful reducing proprieties. It is contained in fresh fruits and not in cooked vegetables, with different percentage depending on the season [93]. It can react with superoxide, hydrogen peroxide, hypochlorite, hydroxyl and peroxyl radicals, and singlet oxygen [94]. Scientific evidence suggests that vitamin C is a required factor in the DNA demethylation process [79] (Table 1).

Boffa et al. (1996) investigated the photoprotective effect of vitamin C in 12 EPP patients with a double-blinded, placebo-controlled, randomized, and crossover study. They found that there was no significant effect of vitamin C on sunlight tolerance [95]. However, an oxidative damage with alteration of lipo peroxidation status not directly correlated to sunlight exposure was reported EPP patients [96]. As for other antioxidant substances, also vitamin C supplementation could be able to improve the oxidative status in order to generate a positive effect on overall health.

Moreover, deficiency of vitamin C has been proposed to contribute to the pathogenesis of PCT [97]. Several studies have shown very low levels of plasma ascorbic acid in patients with active PCT suggesting that a depletion of this molecule could be due to the high intracellular oxidative stress linked to porphyrins and iron accumulation in these patients [98,99,100]. Moreover, the vitamin C deficiency in PCT patients can be explained by the latest observation that it is a powerful nutrigenetic compound able to reduce ferric iron (Fe3+) to ferrous iron (Fe2+), making it available to the catalytic site of the ten-eleven translocation (TET) enzyme. TET enzymes play an important role in DNA demethylation and gene activation, including genes of the Nrf2 antioxidant pathway. Vitamin C administration could perhaps help these patients to improve their antioxidant machinery [101].

### 5.5. Vitamin D

It is a fat-soluble vitamin that plays a key role in the skeletal constitution, cardiovascular disorders, cancers, central nervous system diseases, reproductive diseases, infections, and autoimmune and dermatological disorders [102]. Few naturally occurring foods such as oily fish, liver oil, egg yolks, shiitake mushrooms, liver, or organ meats contain vitamin D in the biologically inactive form [103]. The first step to achieve the active form of vitamin D, 25-hydroxyvitamin D 25-OH (D) occurs in skin epidermal cells after UVB irradiation [104]. For general health, the optimal serum concentration of 25-OH (D) must exceed 30 ng/mL, taking into consideration a variation in the summer season. It is well known that lower latitude and higher amounts of sunshine have been associated with a lower risk of vitamin D deficiency [105,106,107] (Table 1).

Between January and July 2008, vitamin D levels were analyzed in 201 EPP patients in an uncontrolled study. The serum level of 25-OH (D) was 18.32 ng/mL. A slight increase was seen in the summer but not sufficient to reach the normality value in 91% of patients considering 30 ng/mL as the minimum threshold [108]. The same deficiency was reported between June and November 2007 in cross-sectional study on 48 EPP patients in Netherlands [109].

In 2014, a longitudinal controlled prospective cohort study in 53 EPP patients in the United Kingdom versus 109 controls at the same latitude through the seasons confirmed a lack of 25-OH (D). In particular, during the summer season, 18% of control and 47% of EPP showed a low level of vitamin D [110]. The bone mineral density (BMD) was tested in a total of 54 patients in two studies that confirm the prevalence of osteoporosis and osteopenia in EPP patients [111,112].

Based on these observations and the functional role in the prevention of many diseases, vitamin D supplementation as active form cholecalciferol is recommended to prevent vitamin D insufficiency due to sun avoidance in patients affected by cutaneous porphyria. The recommended daily intake during infancy is 400 IU/day; however, after 1-year of age, the recommended daily intake is 600 IU/per day, and after 71 years of age, the recommended daily intake reaches 800 IU/day [113]

## 6. Others Micronutrients and Minerals

Heme biosynthesis also depends on micronutrients important for producing succinyl-CoA for the Krebs (TCA) cycle, which include biotin, lipoic acid, and pantothenic acid. Biotin functions as a prosthetic group in four biotin-dependent carboxylases including propionyl-CoA carboxylase that produces succinyl-CoA. The deficiency for biotin decreases the activity of this enzyme and induces the production of methylcrotonyl-CoA that reacts with glycine [114]. Thus, biotin deficiency results in the depletion of both mitochondrial succinyl-CoA and glycine for heme synthesis. 

Pantothenic acid is the precursor of coenzyme A (CoA) and is important for the production of acetyl-CoA. Lipoic acid is a cofactor for the mitochondrial enzymes pyruvate dehydrogenase (PDH) and α-ketoglutarate dehydrogenase (αKGDH), which produce acetyl-CoA and succinyl-CoA, respectively [115]. A deficiency of either of these may reduce heme biosynthesis through a mechanism similar to that produced by biotin deficiency. 

Riboflavin (FAD) is necessary for protoporphyrinogen oxidase (PPOX) enzyme that catalyzes the formation of protoporphyrin IX while zinc is present in δ-aminolevulinate dehydratase (ALAD). Zinc deficiency, due to the inactivation of ALAD, causes the marked release of oxidants resulting in significant oxidative damage to DNA [116]. Finally, copper plays a role in heme synthesis stimulating the activity of FECH and decreasing the Km for iron [117]. Finally, copper deficiency increases dietary iron absorption [118].

## 7. Conclusions

In conclusion, the dietary pattern observed among porphyria patients was in line with current dietary trends. Usually, porphyria patients are not required to follow a special diet and recommendations are based on prevailing dietary guidelines for the general population. However, in AHP patients, high carbohydrate intake is greatly recommended as a part of a balanced diet that provides all essential nutrients. Although iron deficiency can compromise heme synthesis, iron supplements are not recommended since its excess can be harmful in several porphyrias. Adequate intake of antioxidants should be provided as a part of a balanced diet in addition to the use of antioxidant supplements as beneficial approach to reduce oxidative stress and cellular damage. Moreover, vitamin D supplements should be given to porphyria patients with cutaneous manifestations. However, nutritional recommendations to these individuals for the management of porphyria are still poorly met. It is necessary to translate these recommendations into guidelines, in order to formulate a personalized nutrition for each form of porphyria [119] (Figure 1). To the best of our knowledge, this manuscript represents the first attempt to provide a broad overview about of metabolic and nutritional aspects of these rare diseases.

## Figures and Tables

**Table 1:** Characteristics of vitamins.

Vitamins	Fat-Soluble	Water-Soluble	Deficiency	Main Foods	Nutraceuticals Characteristics
Vitamin B6		x	Rare	Meats, fishery products, offal, cereals, legumes, oilseeds.	⇒ Coenzyme for more than 100 enzyme reactions including glucose metabolism ⇒ Involved in the first step of heme synthesis
Beta-carotene (provit-A)	x		Ultra-rare (more likely to exceed)	In most fruit, cereals, oils, green leafy vegetables.	⇒ Antioxidant proprieties ⇒ Photo-protection in a healthy population ⇒ Toxic dose ≥ 20 mg/day
Vitamin E	x		Uncommon	Nuts, plant seeds, plant oils.	⇒ Antioxidant proprieties ⇒ Synergic action with vitamin C and glutathione reductase
Vitamin C		x	Uncommon	Fresh fruits and vegetables (decreases with cooking and during the seasons).	⇒ Antioxidant proprieties ⇒ Required factor in the DNA demethylation process
Vitamin D	x		Common	Oily fish, liver oil, egg yolks, shiitake mushrooms, liver or organ meats	⇒ Role in the skeletal constitution, ⇒ Optimal dosage in serum to prevent bone disease 30 ng/mL

## Abbreviations

ALA	5-Aminolevulinic acid
ADP	ALA dehydratase deficient porphyria
AHP	Acute hepatic porphyria
AIP	Acute intermittent porphyria
αKGDH	α-Ketoglutarate dehydrogenase
AKT	Protein kinase B
ALAD	5-Aminolevulinate dehydratase
ALAS-1	5-Aminolevulinate synthase 1
ALAS-2	5-aminolevulinate synthase 2
BMD	Bone mineral density
CAT	Antioxidant enzyme catalase
CEP	Congenital erythropoietic porphyria
CREB	cAMP response element-binding factor
DEXA	Dual-energy X-ray absorptiometry
ECP	Erythropoietic cutaneous porphyria
EFSA	European food safety authority
EPP	Erythropoietic protoporphyria
FAD	Riboflavin
FECH	Ferrochelatese
FOXO 1	Forkhead box protein O1
GLR	Glucagon receptor
GR	Glutathione reductase
GSH	Glutathione
GSSG	Glutathione disulfide
HCP	Hereditary coproporphyria
HCV	Hepatitis C virus
HEP	Hepatoerythropoietic porphyria
HFE	Hemochromatosis
HIV	Human immunodeficiency virus
IGF-1	Insulin-like growth factor 1
IRE	Insulin responsive element
IRs	Insulin receptors
MDA	Malondialdehyde
MUFA	Monounsaturated fatty acids
OA	Oxalic acid
25-OH (D)	25-hydroxyvitamin D
PBG	Porphobilinogen
PCT	Porphyria cutanea tarda
PGC-1α	Peroxisomal proliferator-activated cofactor 1α
PHD	Pyruvate dehydrogenase
PI3K	Phosphoinositide 3-kinases
PKA	Protein kinase A
PLP	Pyridoxal-5-phosphate
PPIX	Protoporphyrin IX
PPOX	Protoporphyrinogen oxidase
PUFA	Polyunsaturated fatty acids
SFA	Saturated fatty acids
VP	Variegate porphyria
ZnPP	Zinc protoporphyrin
XLP	X-linked protoporphyria

## References

[1] Thunell S. (2000). Porphyrins, porphyrin metabolism and porphyrias. I. Update. Scand. J. Clin. Lab. Investig..

[2] Kauppinen R. (2005). Porphyrias. Lancet.

[3] Puy H., Gouya L., Deybach J.C. (2010). Porphyrias. Lancet.

[4] Karim Z., Lyoumi S., Nicolas G., Deybach J.C., Gouya L., Puy H. (2015). Porphyrias: A 2015 update. Clin. Res. Hepatol. Gastroenterol..

[5] Wang B., Rudnick S., Cengia B., Bonkovsky H.L. (2019). Acute Hepatic Porphyrias: Review and Recent Progress. Hepatol. Commun..

[6] Bissell D.M., Anderson K.E., Bonkovsky H.L. (2017). Porphyria. N. Engl. J. Med..

[7] Balwani M., Desnick R.J. (2012). The porphyrias: Advances in diagnosis and treatment. Blood.

[8] Egger N.G., Lee C., Anderson K.E., Fernandes J., Saudubray J.M., Van den Berghe G., Walter J.H. (2006). Disorders of Heme Biosynthesis. Inborn Metabolic Diseases.

[9] Welland F.H., Hellman E.S., Gaddis E.M., Collins G., Hunter G.W., Tschudy D.P. (1964). factors affecting the excretion of porphyrin precursors by patients with acute intermittent porphyria. I. The effect of diet. Metabolism.

[10] Storjord E., Dahl J.A., Landsem A., Ludviksen J.K., Karlsen M.B., Karlsen B.O., Brekke O.L. (2019). Lifestyle factors including diet and biochemical biomarkers in acute intermittent porphyria: Results from a case-control study in northern Norway. Mol. Genet. Metab..

[11] Slimani N., Ferrari P., Ocke M., Welch A., Boeing H., Liere M., Pala V., Amiano P., Lagiou A., Mattisson I. (2000). Standardization of the 24-h diet recall calibration method used in the european prospective investigation into cancer and nutrition (EPIC): General concepts and preliminary results. Eur. J. Clin. Nutr..

[12] Romaguera D., Puigros M.A., Palacin C., Pons A., Tur J.A. (2006). Nutritional assessment of patients affected by porphyria variegata. Ann. Nutr. Metab..

[13] Garcia-Diz L., Murcia M.A., Gris J.L., Pons A., Monteagudo C., Martinez-Tome M., Jimenez-Monreal A.M. (2012). Assessing nutritional status of acute intermittent porphyria patients. Eur. J. Clin. Investig..

[14] Fernandez-Miranda C., De La Calle M., Larumbe S., Gomez-Izquierdo T., Porres A., Gomez-Gerique J., de Enriquez S.R. (2000). Lipoprotein abnormalities in patients with asymptomatic acute porphyria. Clin. Chim. Acta.

[15] Muller M.J., Bosy-Westphal A., Kutzner D., Heller M. (2002). Metabolically active components of fat-free mass and resting energy expenditure in humans: Recent lessons from imaging technologies. Obes Rev..

[16] Marsden J.T., Rees D.C. (2014). Urinary excretion of porphyrins, porphobilinogen and delta-aminolaevulinic acid following an attack of acute intermittent porphyria. J. Clin. Pathol..

[17] Robert T.L., Varella L., Meguid M.M. (1994). Nutrition management of acute intermittent porphyria. Nutrition.

[18] Pischik E., Kauppinen R. (2015). An update of clinical management of acute intermittent porphyria. Appl. Clin. Genet..

[19] Anderson K.E., Bloomer J.R., Bonkovsky H.L., Kushner J.P., Pierach C.A., Pimstone N.R., Desnick R.J. (2005). Recommendations for the diagnosis and treatment of the acute porphyrias. Ann. Intern. Med..

[20] Giger U., Meyer U.A. (1981). Induction of delta-aminolevulinate synthase and cytochrome P-450 hemoproteins in hepatocyte culture. Effect of glucose and hormones. J. Biol. Chem..

[21] Tschudy D.P., Welland F.H., Collins A., Hunter G. (1964). The effect of carbohydrate feeding on the induction of delta-aminolevulinic acid synthetase. Metabolism.

[22] Handschin C., Lin J., Rhee J., Peyer A.K., Chin S., Wu P.H., Meyer U.A., Spiegelman B.M. (2005). Nutritional regulation of hepatic heme biosynthesis and porphyria through PGC-1alpha. Cell.

[23] Herzig S., Long F., Jhala U.S., Hedrick S., Quinn R., Bauer A., Rudolph D., Schutz G., Yoon C., Puigserver P. (2001). CREB regulates hepatic gluconeogenesis through the coactivator PGC-1. Nature.

[24] Varone C.L., Giono L.E., Ochoa A., Zakin M.M., Canepa E.T. (1999). Transcriptional regulation of 5-aminolevulinate synthase by phenobarbital and cAMP-dependent protein kinase. Arch. Biochem. Biophys..

[25] Scassa M.E., Guberman A.S., Varone C.L., Canepa E.T. (2001). Phosphatidylinositol 3-kinase and Ras/mitogen-activated protein kinase signaling pathways are required for the regulation of 5-aminolevulinate synthase gene expression by insulin. Exp. Cell Res..

[26] Brunet A., Bonni A., Zigmond M.J., Lin M.Z., Juo P., Hu L.S., Anderson M.J., Arden K.C., Blenis J., Greenberg M.E. (1999). Akt promotes cell survival by phosphorylating and inhibiting a Forkhead transcription factor. Cell.

[27] Nakae J., Kitamura T., Silver D.L., Accili D. (2001). The forkhead transcription factor Foxo1 (Fkhr) confers insulin sensitivity onto glucose-6-phosphatase expression. J. Clin. Investig..

[28] Li D. (2005). PGC-1alpha: Looking behind the sweet treat for porphyria. Cell.

[29] Collantes M., Serrano-Mendioroz I., Benito M., Molinet-Dronda F., Delgado M., Vinaixa M., Sampedro A., de Enriquez S.R., Prieto E., Pozo M.A. (2016). Glucose metabolism during fasting is altered in experimental porphobilinogen deaminase deficiency. Hum. Mol. Genet..

[30] Matkovic L.B., D’Andrea F., Fornes D., de San Martin Viale L.C., Mazzetti M.B. (2011). How porphyrinogenic drugs modeling acute porphyria impair the hormonal status that regulates glucose metabolism. Their relevance in the onset of this disease. Toxicology.

[31] Lithner F. (2002). Beneficial effect of diabetes on acute intermittent porphyria. Diabetes Care.

[32] Storjord E., Dahl J.A., Landsem A., Fure H., Ludviksen J.K., Goldbeck-Wood S., Karlsen B.O., Berg K.S., Mollnes T.E., Nielsen W. (2017). Systemic inflammation in acute intermittent porphyria: A case-control study. Clin. Exp. Immunol..

[33] Delaby C., To-Figueras J., Deybach J.C., Casamitjana R., Puy H., Herrero C. (2009). Role of two nutritional hepatic markers (insulin-like growth factor 1 and transthyretin) in the clinical assessment and follow-up of acute intermittent porphyria patients. J. Intern. Med..

[34] Bernstein L.H., Ingenbleek Y. (2002). Transthyretin: Its response to malnutrition and stress injury. Clinical usefulness and economic implications. Clin. Chem. Lab. Med..

[35] Johnson A.M. (1999). Low levels of plasma proteins: Malnutrition or inflammation?. Clin. Chem. Lab. Med..

[36] Clark M.A., Hentzen B.T., Plank L.D., Hill G.I. (1996). Sequential changes in insulin-like growth factor 1, plasma proteins, and total body protein in severe sepsis and multiple injury. JPEN J. Parenter. Enter. Nutr..

[37] Biolo G., Toigo G., Ciocchi B., Situlin R., Iscra F., Gullo A., Guarnieri G. (1997). Metabolic response to injury and sepsis: Changes in protein metabolism. Nutrition.

[38] Dowman J.K., Gunson B.K., Bramhall S., Badminton M.N., Newsome P.N. (2011). Liver transplantation from donors with acute intermittent porphyria. Ann. Intern. Med..

[39] Soonawalla Z.F., Orug T., Badminton M.N., Elder G.H., Rhodes J.M., Bramhall S.R., Elias E. (2004). Liver transplantation as a cure for acute intermittent porphyria. Lancet.

[40] Stojeba N., Meyer C., Jeanpierre C., Perrot F., Hirth C., Pottecher T., Deybach J.C. (2004). Recovery from a variegate porphyria by a liver transplantation. Liver Transpl..

[41] EFSA Panel on Dietetic Products NaA (2015). Scientific Opinion on Dietary Reference Values for iron1. EFSA J..

[42] Barman-Aksoezen J., Girelli D., Aurizi C., Schneider-Yin X., Campostrini N., Barbieri L., Minder E.I., Biolcati G. (2017). Disturbed iron metabolism in erythropoietic protoporphyria and association of GDF15 and gender with disease severity. J. Inherit. Metab. Dis..

[43] Sampietro M., Fiorelli G., Fargion S. (1999). Iron overload in porphyria cutanea tarda. Haematologica.

[44] Dereure O., Jumez N., Bessis D., Gallix B., Guillot B. (2008). Measurement of liver iron content by magnetic resonance imaging in 20 patients with overt porphyria cutanea tarda before phlebotomy therapy: A prospective study. Acta Derm. Venereol..

[45] Di Pierro E., Brancaleoni V., Granata F. (2016). Advances in understanding the pathogenesis of congenital erythropoietic porphyria. Br. J. Haematol..

[46] Phillips J.D., Bergonia H.A., Reilly C.A., Franklin M.R., Kushner J.P. (2007). A porphomethene inhibitor of uroporphyrinogen decarboxylase causes porphyria cutanea tarda. Proc. Natl. Acad. Sci. USA.

[47] Dabrowska E., Jablonska-Kaszewska I., Falkiewicz B. (2001). Effect of high fiber vegetable-fruit diet on the activity of liver damage and serum iron level in porphyria cutanea tarda (PCT). Med. Sci. Monit..

[48] Singal A.K. (2019). Porphyria cutanea tarda: Recent update. Mol. Genet. Metab..

[49] Mirmiran A., Poli A., Ged C., Schmitt C., Lefebvre T., Manceau H., Daher R., Moulouel B., Peoc’h K., Simonin S. (2020). Phlebotomy as an efficient long-term treatment of congenital erythropoietic porphyria. Haematologica.

[50] Egan D.N., Yang Z., Phillips J., Abkowitz J.L. (2015). Inducing iron deficiency improves erythropoiesis and photosensitivity in congenital erythropoietic porphyria. Blood.

[51] Landefeld C., Kentouche K., Gruhn B., Stauch T., Rossler S., Schuppan D., Whatley S.D., Beck J.F., Stolzel U. (2016). X-linked protoporphyria: Iron supplementation improves protoporphyrin overload, liver damage and anaemia. Br. J. Haematol..

[52] Balwani M. (2019). Erythropoietic Protoporphyria and X-Linked Protoporphyria: Pathophysiology, genetics, clinical manifestations, and management. Mol. Genet. Metab..

[53] Minder E.I., Barman-Aksozen J. (2015). Iron and erythropoietic porphyrias. Blood.

[54] Barman-Aksozen J., Halloy F., Iyer P.S., Schumperli D., Minder A.E., Hall J., Minder E.I., Schneider-Yin X. (2019). Delta-aminolevulinic acid synthase 2 expression in combination with iron as modifiers of disease severity in erythropoietic protoporphyria. Mol. Genet. Metab..

[55] Gordeuk V.R., Brittenham G.M., Hawkins C.W., Mukhtar H., Bickers D.R. (1986). Iron therapy for hepatic dysfunction in erythropoietic protoporphyria. Ann. Intern. Med..

[56] McClements B.M., Bingham A., Callender M.E., Trimble E.R. (1990). Erythropoietic protoporphyria and iron therapy. Br. J. Dermatol..

[57] Milligan A., Graham-Brown R.A., Sarkany I., Baker H. (1988). Erythropoietic protoporphyria exacerbated by oral iron therapy. Br. J. Dermatol..

[58] Holme S.A., Thomas C.L., Whatley S.D., Bentley D.P., Anstey A.V., Badminton M.N. (2007). Symptomatic response of erythropoietic protoporphyria to iron supplementation. J. Am. Acad. Dermatol..

[59] Hemminger A., Wills B.K. (2020). Vitamin B6 Toxicity.

[60] Linkswiler H. (1967). Biochemical and physiological changes in vitamin B6 deficiency. Am. J. Clin. Nutr..

[61] Hamfelt A., Wetterberg L. (1969). Pyridoxal phosphate in acute intermittent porphyria. Ann. N. Y. Acad. Sci..

[62] Elder T.D., Mengel C.E. (1966). Effect of pyridoxine deficiency on porphyrin precursor excretion in acute intermittent porphyria. Am. J. Med..

[63] Mydlik M., Derzsiova K. (2010). Vitamin B6 and oxalic acid in clinical nephrology. J. Ren. Nutr..

[64] Scheer J.B., Mackey A.D., Gregory J.F. (2005). Activities of hepatic cytosolic and mitochondrial forms of serine hydroxymethyltransferase and hepatic glycine concentration are affected by vitamin B-6 intake in rats. J. Nutr..

[65] Thompson J.S., Richardson K.E. (1967). Isolation and characterization of an L-alanine: Glyoxylate aminotransferase from human liver. J. Biol. Chem..

[66] To-Figueras J., Lopez R.M., Deulofeu R., Herrero C. (2010). Preliminary report: Hyperhomocysteinemia in patients with acute intermittent porphyria. Metabolism.

[67] Chabner B.A., Stein J.A., Tschudy D.P. (1970). Effect on dietary pyridoxine deficiency on experimental porphyria. Metabolism.

[68] Balic A., Mokos M. (2019). Do We Utilize Our Knowledge of the Skin Protective Effects of Carotenoids Enough?. Antioxidants.

[69] Mathews-Roth M.M., Pathak M.A., Fitzpatrick T.B., Harber L.H., Kass E.H. (1977). Beta carotene therapy for erythropoietic protoporphyria and other photosensitivity diseases. Arch. Dermatol..

[70] Mathews-Roth M.M. (1986). Beta-carotene therapy for erythropoietic protoporphyria and other photosensitivity diseases. Biochimie.

[71] Alemzadeh R., Feehan T. (2004). Variable effects of beta-carotene therapy in a child with erythropoietic protoporphyria. Eur. J. Pediatr..

[72] Minder E.I., Schneider-Yin X., Steurer J., Bachmann L.M. (2009). A systematic review of treatment options for dermal photosensitivity in erythropoietic protoporphyria. Cell. Mol. Biol. (Noisy -le-grand).

[73] Balwani M., Desnick R. (1993). X-Linked Protoporphyria.

[74] Balwani M., Bloomer J., Desnick R. (1993). Erythropoietic Protoporphyria, Autosomal Recessive.

[75] Tintle S., Alikhan A., Horner M.E., Hand J.L., Davis D.M. (2014). Cutaneous porphyrias part II: Treatment strategies. Int. J. Dermatol..

[76] Bafteh P.R., Siegesmund M., Hanneken S., Neumann N.J. (2012). Protective effects of beta-carotene and melanin against protoporphyrine IX-induced phototoxicity in the photo hen’s egg test. Photodermatol. Photoimmunol. Photomed..

[77] Rocchi E., Stella A.M., Cassanelli M., Borghi A., Nardella N., Seium Y., Casalgrandi G. (1995). Liposoluble vitamins and naturally occurring carotenoids in porphyria cutanea tarda. Eur. J. Clin. Investig..

[78] EFSA Panel on Food Additives and Nutrient Sources added to Food (ANS) (2012). Scientific Opinion on the re-evaluation of Mixed Carotenes (E 160a (i)) and beta-Carotene (E 160a (ii)) as a food additive. EFSA J..

[79] Beetch M., Harandi-Zadeh S., Shen K., Lubecka K., Kitts D.D., O’Hagan H.M., Stefanska B. (2020). Dietary antioxidants remodel DNA methylation patterns in chronic disease. Br. J. Pharmacol..

[80] Jeanes Y.M., Hall W.L., Ellard S., Lee E., Lodge J.K. (2004). The absorption of vitamin E is influenced by the amount of fat in a meal and the food matrix. Br. J. Nutr..

[81] Monteiro H.P., Abdalla D.S., Faljoni-Alario A., Bechara E.J. (1986). Generation of active oxygen species during coupled autoxidation of oxyhemoglobin and delta-aminolevulinic acid. Biochim. Biophys. Acta.

[82] Thunell S., Andersson C., Carlmark B., Floderus Y., Gronqvist S.O., Harper P., Henrichson A., Lindh U. (1995). Markers for vulnerability in acute porphyria. A hypothesis paper. Eur. J. Clin. Chem. Clin. Biochem..

[83] Rocchi E., Casalgrandi G., Masini A., Giovannini F., Ceccarelli D., Ferrali M., Marchini S., Ventura E. (1999). Circulating pro- and antioxidant factors in iron and porphyrin metabolism disorders. Ital. J. Gastroenterol. Hepatol..

[84] Ferrer M.D., Tauler P., Sureda A., Palacin C., Tur J.A., Pons A. (2010). Variegate porphyria induces plasma and neutrophil oxidative stress: Effects of dietary supplementation with vitamins E and C. Br. J. Nutr..

[85] Rocchi E., Ventura P., Ronzoni A., Rosa M.C., Gozzi C., Marri L., Casalgrandi G., Cappellini M.D. (2004). Pro-oxidant and antioxidant factors in acute intermittent porphyria: Family studies. J. Inherit. Metab. Dis..

[86] Pinelli A., Trivulzio S., Tomasoni L., Bertolini B., Pinelli G. (2002). High-dose vitamin E lowers urine porphyrin levels in patients affected by porphyria cutanea tarda. Pharmacol. Res..

[87] Szekely E., Vereckei A., Almasi A., Rapavi E., Tasnadi G., Varnai K., Pallai Z., Lugasi A., Blazovics A. (2007). Effects of vitamin E administration on the hemorheological status and redox homeostasis of patients with porphyria cutanea tarda treated with phlebotomy. Clin. Hemorheol. Microcirc..

[88] Thunell S., Andersson D., Harper P., Henrichson A., Floderus Y., Lindh U. (1997). Effects of administration of antioxidants in acute intermittent porphyria. Eur. J. Clin. Chem. Clin. Biochem..

[89] Adjarov D., Ribarova F., Koytcheva N., Shishkov S., Ivanova A., Antonov K. (2001). Impaired antioxidant status in porphyria cutanea tarda. Acta Medica Bulg..

[90] Johnson J.A., Fusaro R.M. (1973). Possible use of vitamins C and-or E in erythropoietic protoporphyria. JAMA.

[91] Fryer M.J. (1993). Evidence for the photoprotective effects of vitamin E. Photochem. Photobiol..

[92] Komatsu H., Ishii K., Imamura K., Maruyama K., Yonei Y., Masuda H., Tsuchihashi T., Sajima Y. (2000). A case of erythropoietic protoporphyria with liver cirrhosis suggesting a therapeutic value of supplementation with alpha-tocopherol. Hepatol. Res..

[93] Phillips K.M., Tarrago-Trani M.T., McGinty R.C., Rasor A.S., Haytowitz D.B., Pehrsson P.R. (2018). Seasonal variability of the vitamin C content of fresh fruits and vegetables in a local retail market. J. Sci. Food Agric..

[94] Traber M.G., Stevens J.F. (2011). Vitamins C and E: Beneficial effects from a mechanistic perspective. Free Radic. Biol. Med..

[95] Boffa M.J., Ead R.D., Reed P., Weinkove C. (1996). A double-blind, placebo-controlled, crossover trial of oral vitamin C in erythropoietic protoporphyria. Photodermatol. Photoimmunol. Photomed..

[96] Granata F., Duca L., Graziadei G., Brancaleoni V., Missineo P., De Luca G., Fustinoni S., Di Pierro E. (2019). Inflammatory involvement into phototoxic reaction in erythropoietic protoporphyria (EPP) patients. Immunol. Res..

[97] Wang K., Jiang H., Li W., Qiang M., Dong T., Li H. (2018). Role of Vitamin C in Skin Diseases. Front. Physiol..

[98] Percy V.A., Naidoo D., Joubert S.M., Pegoraro R.J. (1975). Ascorbate status of patients with porphyria cutanea tarda symptomatica and its effect on porphyrin metabolism. S. Afr. J. Med. Sci..

[99] Sinclair P.R., Gorman N., Shedlofsky S.I., Honsinger C.P., Sinclair J.F., Karagas M.R., Anderson K.E. (1997). Ascorbic acid deficiency in porphyria cutanea tarda. J. Lab. Clin. Med..

[100] Fedeles F., Murphy M., Rothe M.J., Grant-Kels J.M. (2010). Nutrition and bullous skin diseases. Clin. Dermatol..

[101] Duarte T.L., Lunec J. (2005). Review: When is an antioxidant not an antioxidant? A review of novel actions and reactions of vitamin C. Free Radic. Res..

[102] Kechichian E., Ezzedine K. (2018). Vitamin D and the Skin: An Update for Dermatologists. Am. J. Clin. Dermatol..

[103] Chang S.W., Lee H.C. (2019). Vitamin D and health—The missing vitamin in humans. Pediatr. Neonatol..

[104] Ross A.C., Manson J.E., Abrams S.A., Aloia J.F., Brannon P.M., Clinton S.K., Durazo-Arvizu R.A., Gallagher J.C., Gallo R.L., Jones G. (2011). The 2011 Dietary Reference Intakes for Calcium and Vitamin D: What dietetics practitioners need to know. J. Am. Diet. Assoc..

[105] Major J.M., Graubard B.I., Dodd K.W., Iwan A., Alexander B.H., Linet M.S., Freedman D.M. (2013). Variability and reproducibility of circulating vitamin D in a nationwide U.S. population. J. Clin. Endocrinol. Metab..

[106] Holick M.F. (2008). The vitamin D deficiency pandemic and consequences for nonskeletal health: Mechanisms of action. Mol. Asp. Med..

[107] Touvier M., Deschasaux M., Montourcy M., Sutton A., Charnaux N., Kesse-Guyot E., Assmann K.E., Fezeu L., Latino-Martel P., Druesne-Pecollo N. (2015). Determinants of vitamin D status in Caucasian adults: Influence of sun exposure, dietary intake, sociodemographic, lifestyle, anthropometric, and genetic factors. J. Investig. Dermatol..

[108] Holme S.A., Anstey A.V., Badminton M.N., Elder G.H. (2008). Serum 25-hydroxyvitamin D in erythropoietic protoporphyria. Br. J. Dermatol..

[109] Spelt J.M., de Rooij F.W., Wilson J.H., Zandbergen A.A. (2010). Vitamin D deficiency in patients with erythropoietic protoporphyria. J. Inherit. Metab. Dis..

[110] Rhodes L.E., Webb A.R., Berry J.L., Felton S.J., Marjanovic E.J., Wilkinson J.D., Vail A., Kift R. (2014). Sunlight exposure behaviour and vitamin D status in photosensitive patients: Longitudinal comparative study with healthy individuals at U.K. latitude. Br. J. Dermatol..

[111] Allo G., del Carmen Garrido-Astray M., Mendez M., De Salamanca R.E., Martinez G., Hawkins F. (2013). Bone mineral density and vitamin D levels in erythropoietic protoporphyria. Endocrine.

[112] Biewenga M., Matawlie R.H.S., Friesema E.C.H., Koole-Lesuis H., Langeveld M., Wilson J.H.P., Langendonk J.G. (2017). Osteoporosis in patients with erythropoietic protoporphyria. Br. J. Dermatol..

[113] Holick M.F., Binkley N.C., Bischoff-Ferrari H.A., Gordon C.M., Hanley D.A., Heaney R.P., Murad M.H., Weaver C.M. (2011). Evaluation, treatment, and prevention of vitamin D deficiency: An Endocrine Society clinical practice guideline. J. Clin. Endocrinol. Metab..

[114] Atamna H. (2004). Heme, iron, and the mitochondrial decay of ageing. Ageing Res. Rev..

[115] Gibson G.E., Park L.C., Sheu K.F., Blass J.P., Calingasan N.Y. (2000). The alpha-ketoglutarate dehydrogenase complex in neurodegeneration. Neurochem. Int..

[116] Ho E., Courtemanche C., Ames B.N. (2003). Zinc deficiency induces oxidative DNA damage and increases p53 expression in human lung fibroblasts. J. Nutr..

[117] Wagner G.S., Tephly T.R. (1975). A possible role of copper in the regulation of heme biosynthesis through ferrochelatase. Adv. Exp. Med. Biol..

[118] Thomas C., Oates P.S. (2003). Copper deficiency increases iron absorption in the rat. Am. J. Physiol. Gastrointest. Liver Physiol..

[119] Bashiardes S., Abdeen S.K., Elinav E. (2019). Personalized Nutrition: Are We There Yet?. J. Pediatr. Gastroenterol. Nutr..

